# Spatial Distribution of Epigenetic Modifications in *Brachypodium distachyon* Embryos during Seed Maturation and Germination

**DOI:** 10.1371/journal.pone.0101246

**Published:** 2014-07-09

**Authors:** Elzbieta Wolny, Agnieszka Braszewska-Zalewska, Robert Hasterok

**Affiliations:** Department of Plant Anatomy and Cytology, Faculty of Biology and Environmental Protection, University of Silesia in Katowice, Katowice, Poland; University of Massachusetts Amherst, United States of America

## Abstract

Seed development involves a plethora of spatially and temporally synchronised genetic and epigenetic processes. Although it has been shown that epigenetic mechanisms, such as DNA methylation and chromatin remodelling, act on a large number of genes during seed development and germination, to date the global levels of histone modifications have not been studied in a tissue-specific manner in plant embryos. In this study we analysed the distribution of three epigenetic markers, i.e. H4K5ac, H3K4me2 and H3K4me1 in ‘matured’, ‘dry’ and ‘germinating’ embryos of a model grass, *Brachypodium distachyon* (Brachypodium). Our results indicate that the abundance of these modifications differs considerably in various organs and tissues of the three types of Brachypodium embryos. Embryos from matured seeds were characterised by the highest level of H4K5ac in RAM and epithelial cells of the scutellum, whereas this modification was not observed in the coleorhiza. In this type of embryos H3K4me2 was most evident in epithelial cells of the scutellum. In ‘dry’ embryos H4K5ac was highest in the coleorhiza but was not present in the nuclei of the scutellum. H3K4me1 was the most elevated in the coleoptile but absent from the coleorhiza, whereas H3K4me2 was the most prominent in leaf primordia and RAM. In embryos from germinating seeds H4K5ac was the most evident in the scutellum but not present in the coleoptile, similarly H3K4me1 was the highest in the scutellum and very low in the coleoptile, while the highest level of H3K4me2 was observed in the coleoptile and the lowest in the coleorhiza. The distinct patterns of epigenetic modifications that were observed may be involved in the switch of the gene expression profiles in specific organs of the developing embryo and may be linked with the physiological changes that accompany seed desiccation, imbibition and germination.

## Introduction

The seed is an important stage in the life cycle of higher plants. It is the dispersal unit of a plant that is able to survive the period between seed maturation and germination [1]. Seed development can be divided into two distinct stages–morphogenesis and maturation. It is initiated by embryogenesis in which a mature embryo develops from a single fertilised cell through a series of processes. Following morphogenesis, the developing seed enters the maturation stage [2]. This stage commences with the transition phase during which the switch from maternal to filial control occurs. The seed then undergoes a period of embryo growth, the so-called reserve accumulation, reorganisation of metabolism and synthesis of storage compounds [2]. Maturation ends with the desiccation phase after which the embryo enters into a quiescent state [3]. This stage is associated with a major loss of water, which leads to a dry seed in preparation for the quiescent period, dormancy and thereafter germination. Three stages can be distinguished during germination: (i) seed imbibition and reinitiating metabolic processes; (ii) limited water uptake; (iii) increased water uptake and the emergence of the radicle [4], [5].

Seed dormancy is considered to be the failure of an intact viable seed to complete germination within a specified period of time under any combination of normal physical environmental factors that are favourable for its germination. It is determined by genetic factors that have an important environmental influence, which is mediated, at least in part, by the plant hormones, abscisic acid (ABA) and gibberellins (GA) [6], [7]. ABA accumulation prevents premature germination during embryo maturation, establishes and later maintains seed dormancy [8]. Loss of dormancy through after-ripening is associated with ABA turnover [9]. GA, on the other hand, accumulates during cold stratification and is required for *Arabidopsis thaliana* (Arabidopsis) seed germination [10], [11]. According to the hormone balance theory, the antagonism between these two hormones regulates seed dormancy, cold stratification, after-ripening and germination [12]. A quiescent, dormant seed consists of approximately 10% water [13].

Dry seeds represent the transitional state between an embryo and seedling. At crucial points during the plant life cycle, when the cells or tissues transform towards a new fate or function, the chromatin undergoes structural changes in its organisation. For spermatophytes, the transition from a dormant to a non-dormant state of the seed is of major importance for successfully establishing a new generation [14]. During the transition phase, the genes that control the ’new’ state need to be activated, while the genes required for the ’old’ state must be repressed [15]. Transcriptomic analyses have revealed a strict spatial and temporal regulation of gene expression during the dormancy to germination transition [7], [16]. Gene *expression can* be influenced by *epigenetic modifications, such as* DNA methylation and histone modifications. N-terminal histone tails are subjected to various posttranslational covalent modifications, including acetylation, methylation, phosphorylation, ubiquitination, ribosylation, glycosylation and sumoylation [17]. Acetylated histones are the hallmarks of transcriptionally active chromatin regions. The histone H3 methylation of lysine K4, K36 and K79 correlates with active transcription, while the methylation of K9, K27 and H4K20 as well as DNA methylation are modifications that are typical for silenced chromatin [18].

Several studies have been devoted to the ultrastructural description of quiescent embryo cells and their changes during germination. The most spectacular ones generally occur in cell nuclei. A high degree of chromatin condensation is established in the nuclei of embryo cells at the end of seed maturation when embryo dehydration occurs [19]. Chromatin compaction has been proposed to contribute to gene regulation by allowing differential accessibility of DNA for the transcription machinery [15], [20]. Recently, van Zanten et al. [21] analysed the nuclear morphology and chromatin organisation in maturing and dry seeds of Arabidopsis at the microscopic level using 4′,6-diamidino-2-phenylindole (DAPI) staining and revealed that there is a major decrease in nuclear size in a maturing seed.

The monocot embryo represents the bilateral symmetry that is established during early embryogenesis in the transition phase. Auxins are the endogenous factors that appear to be involved in the shift from a radial to a bilateral symmetry [22], [23]. The embryo of monocots contains a single terminal cotyledon and the shoot apex (apical meristem) is situated lateral to it. The dicots, in contrast, possess an embryo with a terminal epicotyl (shoot apex) that is subtended laterally by two oppositely placed cotyledons. Members of the grass family have a specialised cotyledon, a scutellum, which plays a pivotal role in the mobilisation of reserve proteins during germination. The embryo of a grass seed is enclosed by a coleorhiza, which covers the root apical meristem (RAM), and a coleoptile, which covers the shoot apical meristem (SAM). Recently, comprehensive analyses of the embryo and grain development of *Brachypodium distachyon* (Brachypodium) were preformed [24]–[27]. The Brachypodium embryo is almost identical to the embryos of barley and wheat anatomically. This wild grass is a model for temperate cereals and forage grasses due to its numerous valuable features, such as its small stature and genome size and short life cycle, which make it suitable for laboratory research [28]–[30]. Recently, Barrero et al. [24] proposed Brachypodium as an ideal model for studies of grain dormancy in grasses and recommended that it be used to identify new strategies for increasing grain dormancy in crop species.

In this study we analyse and discuss the anatomy of the Brachypodium embryo, starch accumulation and epigenetic modifications in ‘matured’, ‘dry’ and ‘germinating’ embryos of the Bd21 reference genotype. To the best of our knowledge this is the first study in plants that links some physiological and developmental aspects of Brachypodium embryos with their epigenetic status that is analysed in a topographical context. We chose three typical markers of euchromatin, which are known to be involved in processes such as DNA replication (H4K5ac) and transcription (H3K4me1,2), to determine if these modifications are present in the embryos at different stages of seed development. Particular attention was paid to dry seeds, as it was assumed that many key processes associated with germination may occur there [4], and chromatin of ‘dry’ embryos may exhibit a transcriptionally active state with high levels of euchromatin-specific markers. There is much data on the dynamics of the turnover of epigenetic modifications during plant development. Through the developmental regulation of these epigenetic mechanisms, plants undergo epigenetic reprogramming in various cell types and developmental stages. The distinct global epigenetic patterns that are revealed in this study may be involved in the switch of the gene expression profiles in specific organs of the developing embryo and may link with the physiological changes that accompany seed desiccation, imbibition and germination.

## Materials and Methods

### Plant material and slide preparation

**Figure 1:**
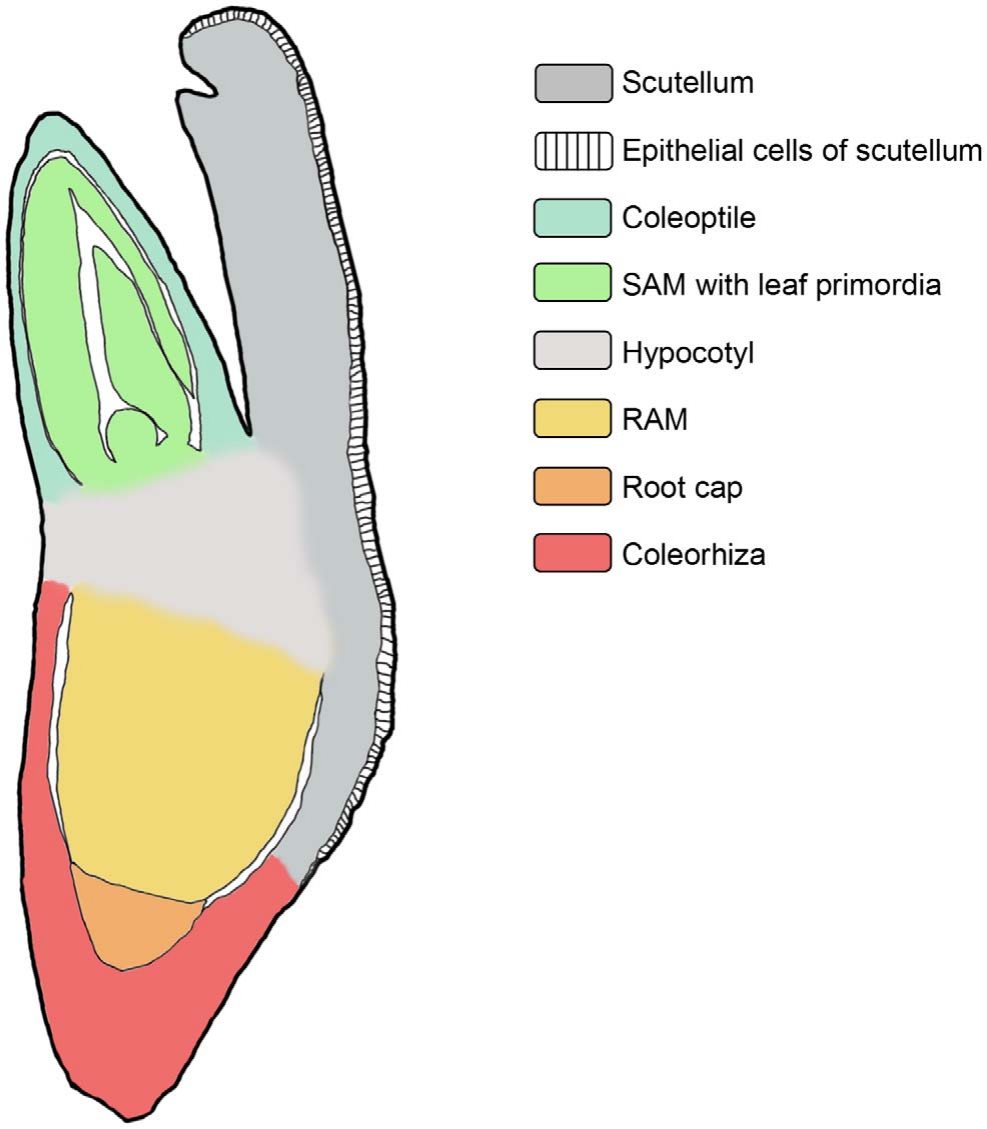
Schematic representation of a longitudinal cross section through a Brachypodium embryo with specific organ tissues marked.

Seeds of *B. distachyon* line Bd21 (Brachypodium) were sown in pots with soil mixed with vermiculite (3∶1 w/w). The plants were grown in a greenhouse at 20±1°C and illuminated by lamps emitting white light with an intensity of 10,000 lx and a 16/8 h light/dark photoperiod. In order to induce synchronised flowering, four-week-old plants were subjected to vernalisation for four weeks at 4°C. After two weeks the plants started to flower. Three types of embryos were used–those obtained from matured, dried and germinating seeds. For simplification, we will refer to them as ‘matured’, ‘dry’ and ‘germinating’ embryos, respectively. ‘Matured’ embryos were selected from grains at 30 days after fertilisation (DAF), ‘dry’ embryos were derived from dry (three months old) seeds. ‘Germinating’ embryos originated from seeds that were placed in a Petri dish on moist filter paper at RT in the dark for 12 h (Figure 1). Whole seeds including the appropriate embryos were fixed in 4% formaldehyde in PBS and placed in a vacuum desiccator for two hours. The material was washed in PBS to remove the fixative. Embryos were manually excised from seeds and dehydrated in a graded ethanol series in a PBS solution (30%, 50%, 70% and 90%) for 30 minutes each and in 99.8% twice for 30 minutes. The embedding medium [31] was prepared from polyethylene glycol 400 distearate and 1-hexadecanol (9/1 w/w). Embedding was done at 37°C in a graded wax/ethanol series (1*/*2, 1*/*1, 2*/*1 v/v) 24 h each followed by one change of pure wax for 24 h. Embryos were then put into embedding moulds and left to polymerise overnight at room temperature. Embryos were sectioned on a microtome (Leica RM 2145) to five *µ*m-thick tissue sections, placed on poly-L-lysine-coated slides and stretched by the addition of a small drop of water. Slides were allowed to dry overnight at RT. After de-embedding three times for 10 min in 99.8% ethanol and rehydrated in ethanol/PBS for 10 min each step (90%, 70%, 50%, 30% v/v and PBS only), the sections were used for immunostaining.

### Immunostaining procedures and PAS staining

The immunostaining method used was as described by Braszewska-Zalewska et al. [32], [33]. The following rabbit monoclonal and polyclonal antibodies against modified histones and DNA were used: anti-monomethyl histone H3 at lysine 4 (1∶100 dilution in 1×PBS, Millipore cat. No 07-436), anti-dimethyl histone H3 at lysine 4 (1∶100 dilution in 1% BSA in 1×PBS; Upstate, Cat. no. 07-030 and Millipore, Cat. no. 07-790) and anti-acetyl histone H4 at lysine 5 (1∶100; Millipore, Cat. no. 04-118). Alexa Fluor 488 goat anti-rabbit IgG (Invitrogen, Molecular Probes, Cat. no. A-11008) was applied as the secondary antibody. As negative controls for immunostaining procedures, detection without a primary antibody was adopted. The expected result of such a procedure is DAPI fluorescence only in the nuclei and autofluorescence of the cell walls. Additionally, to rule out unspecific fluorescence related to the fixation and/or wax procedure, control slides were prepared without both primary and/or secondary antibodies as well as without DAPI: only BSA and PBS buffer were used resulting in autofluorescence in cell walls only.

For the PAS staining procedure slides with tissue sections were de-embedded three times for 10 min in 99.8% ethanol and rehydrated in ethanol/distilled water for 10 min each step (90%, 70%, 50%, 30% v/v, distilled water). Slides were then oxidised in a 0.5% periodic acid solution for 60 minutes, rinsed in distilled water and placed in a Schiff reagent (Merck) for 30 minutes. Stained slides were washed in distilled water, dehydrated in a graded ethanol series in distilled water (30%, 50%, 70%, 90% and 99.8%) for one minute each and embedded in a mounting medium (Euparal).

### Image acquisition, processing and quantitative analysis

Images of embryo cross sections were registered using a high-content screening system (Scan∧R, Olympus) based on an Olympus IX81 wide-field epifluorescence microscope equipped with an ORCA-ER CCD camera (Hammamatsu Photonics) and an MT20 illumination system based on a Xenon-mercury lamp (150W) as described by Braszewska-Zalewska et al. [33], [34]. Images of PAS stained cross sections were obtained using a NICON Eclipse *Ni* microscope.

## Results

**Figure 2:**
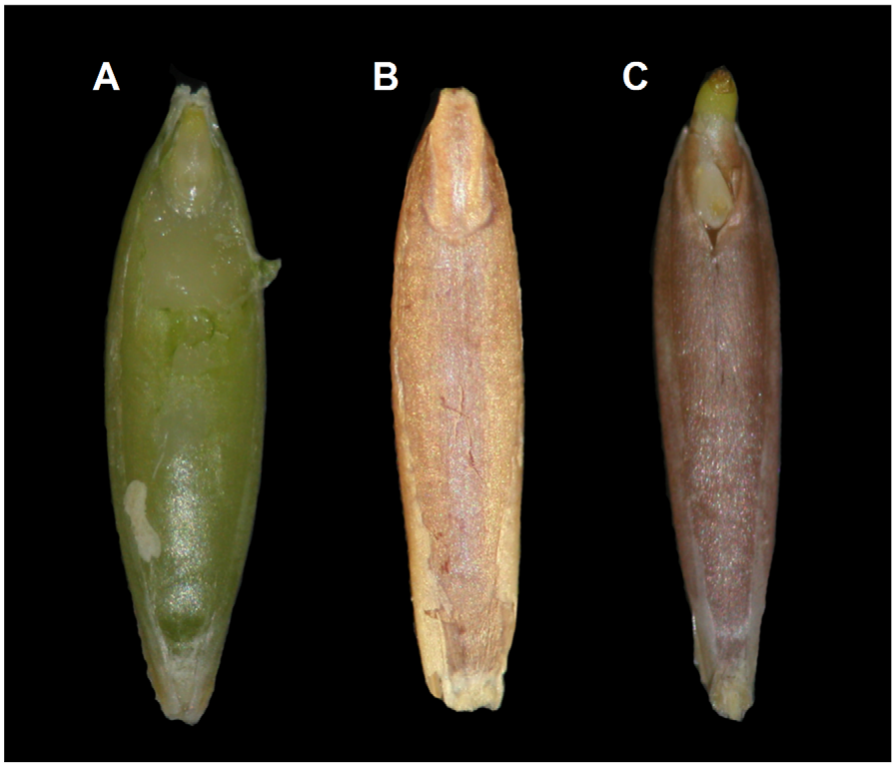
Seeds of Brachypodium with a ‘matured’ (A), ‘dry’ (B) and ‘germinating’ (C) embryo. Bar: 1 mm.

**Figure 3:**
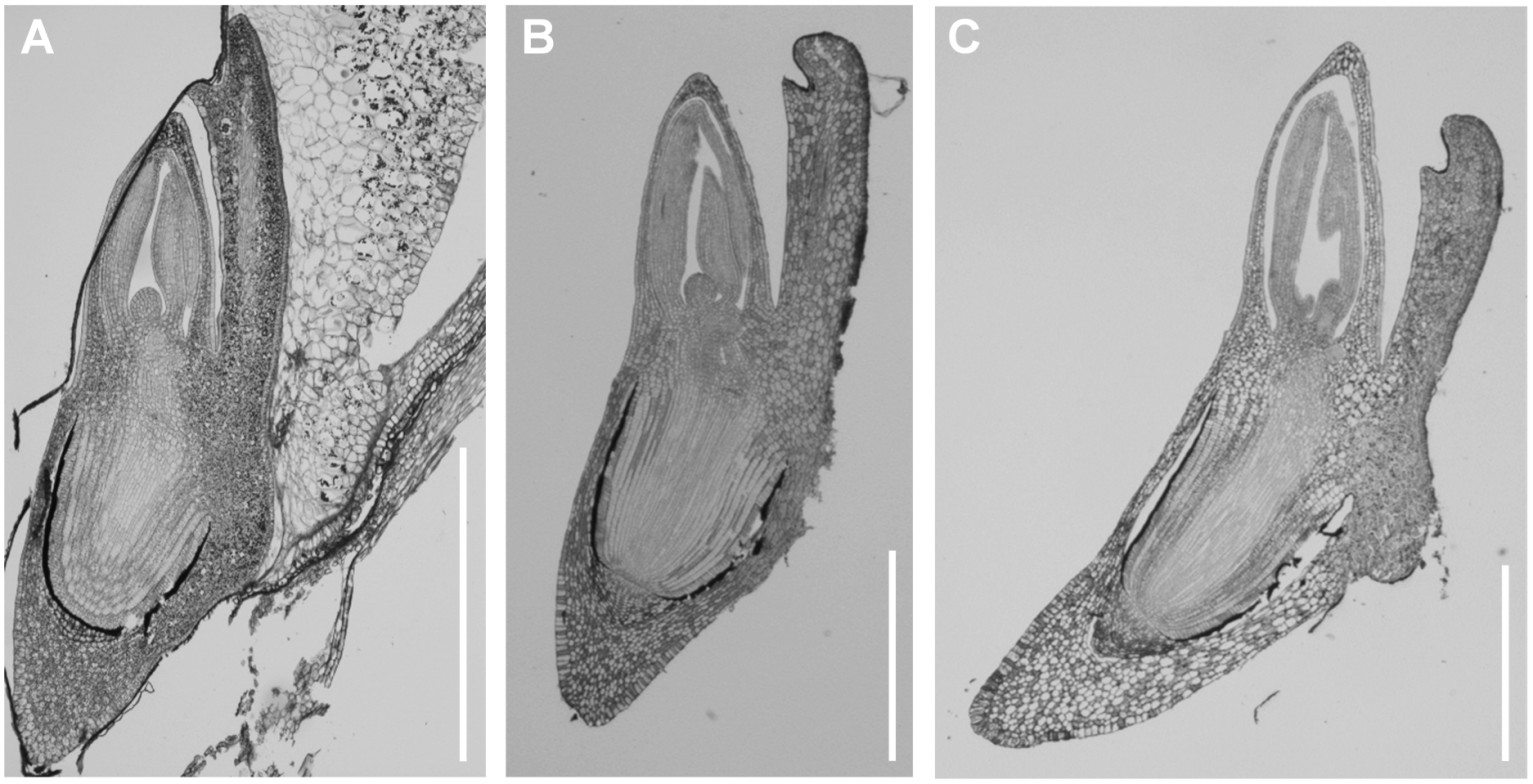
Longitudinal cross sections through the whole ‘matured’ (A), ‘dry’ (B) and ‘germinating’ (C) Brachypodium embryo. Bar: 0.5 mm.

**Figure 4:**
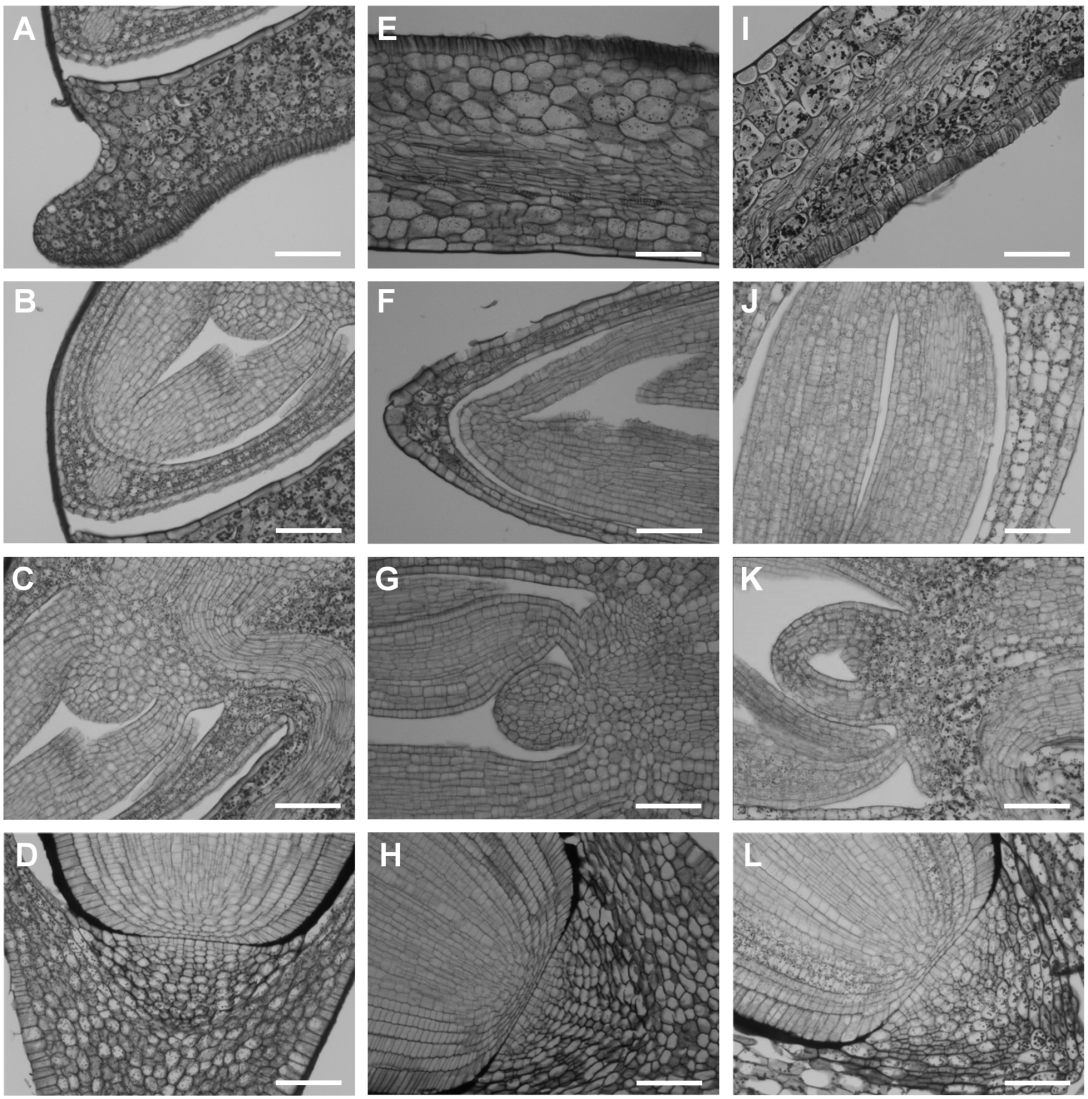
Starch accumulation in ‘matured’ (A–D), ‘dry’ (E–H) and ‘germinating’ (I–L) Brachypodium embryos detected by PAS reaction. Cross sections through the scutellum (A, E, I), coleoptile and SAM with leaf primordia (B, F, J), RAM (C, G, K), the root cap and coleorhiza (D, H, L). Bar: 50 µm.

**Figure 5:**
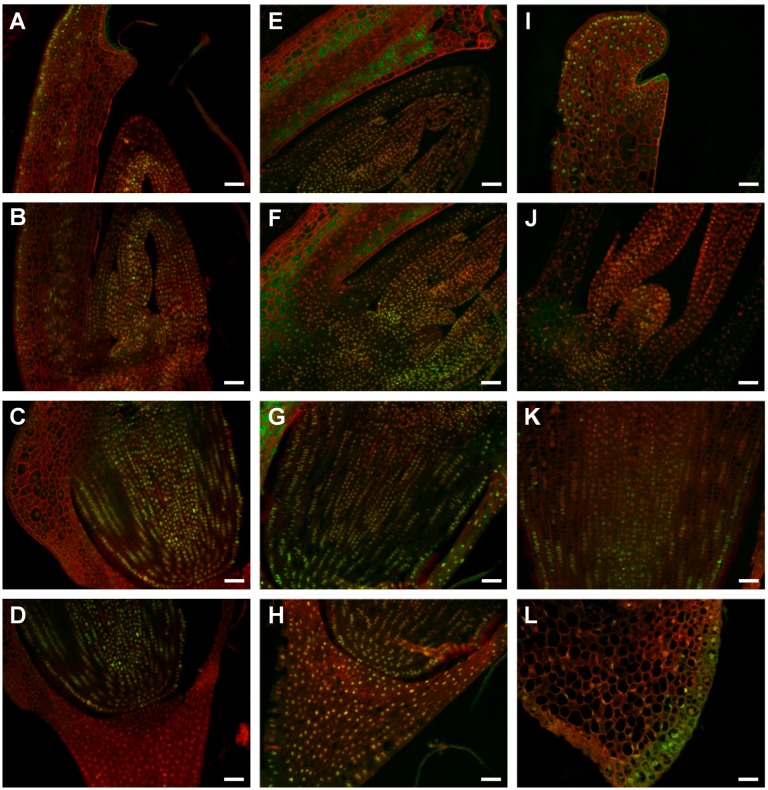
The immunodetection of H4K5ac in ‘matured’ (A–D), ‘dry’ (E–H) and ‘germinating’ (I–L) Brachypodium embryos. Cross sections through the scutellum (**A, I**), the scutellum, coleoptile and leaf primordia (**E**), the SAM with leaf primordia (**B, F, J**), the RAM (**C, G, K**), the distal part of RAM, the root cap and coleorhiza (**D, H**) and the coleorhiza (**L**). Bar: 50 µm. Enlargements of selected cross sections are provided (Figure S1).

**Figure 6:**
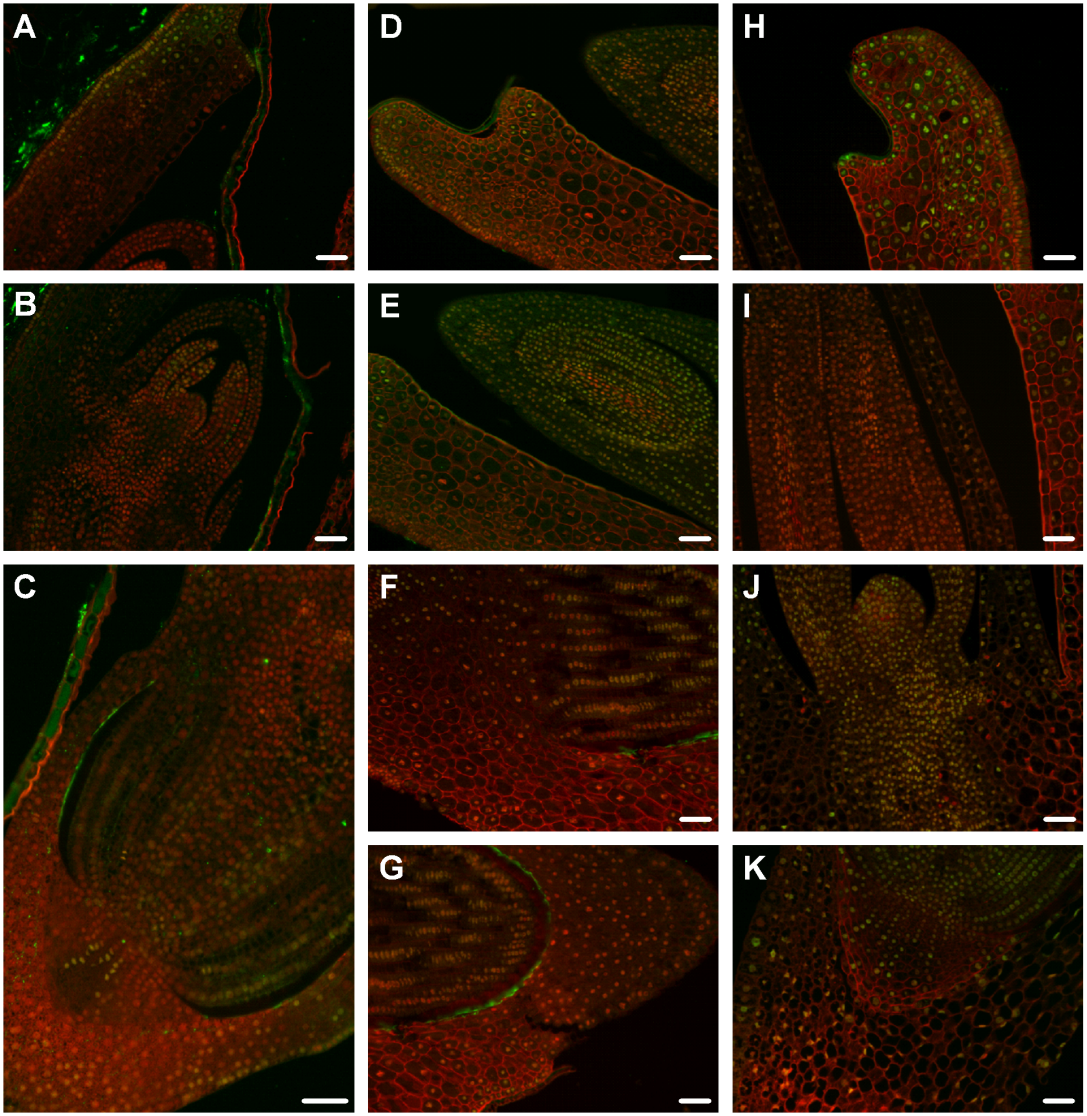
The immunodetection of H3K4me1 in ‘matured’ (A–C), ‘dry’ (D–G) and ‘germinating’ (H–K) Brachypodium embryos. Cross sections through the scutellum (**A, D, H**), the coleoptile and SAM with leaf primordia (**B**), the coleoptile and leaf primordia (**E, I**), the SAM (**J**), the RAM, the root cap and coleorhiza (**C, K**), RAM (**F**), the distal part of RAM and the coleorhiza (**G**). Bar: 50 µm.

**Figure 7:**
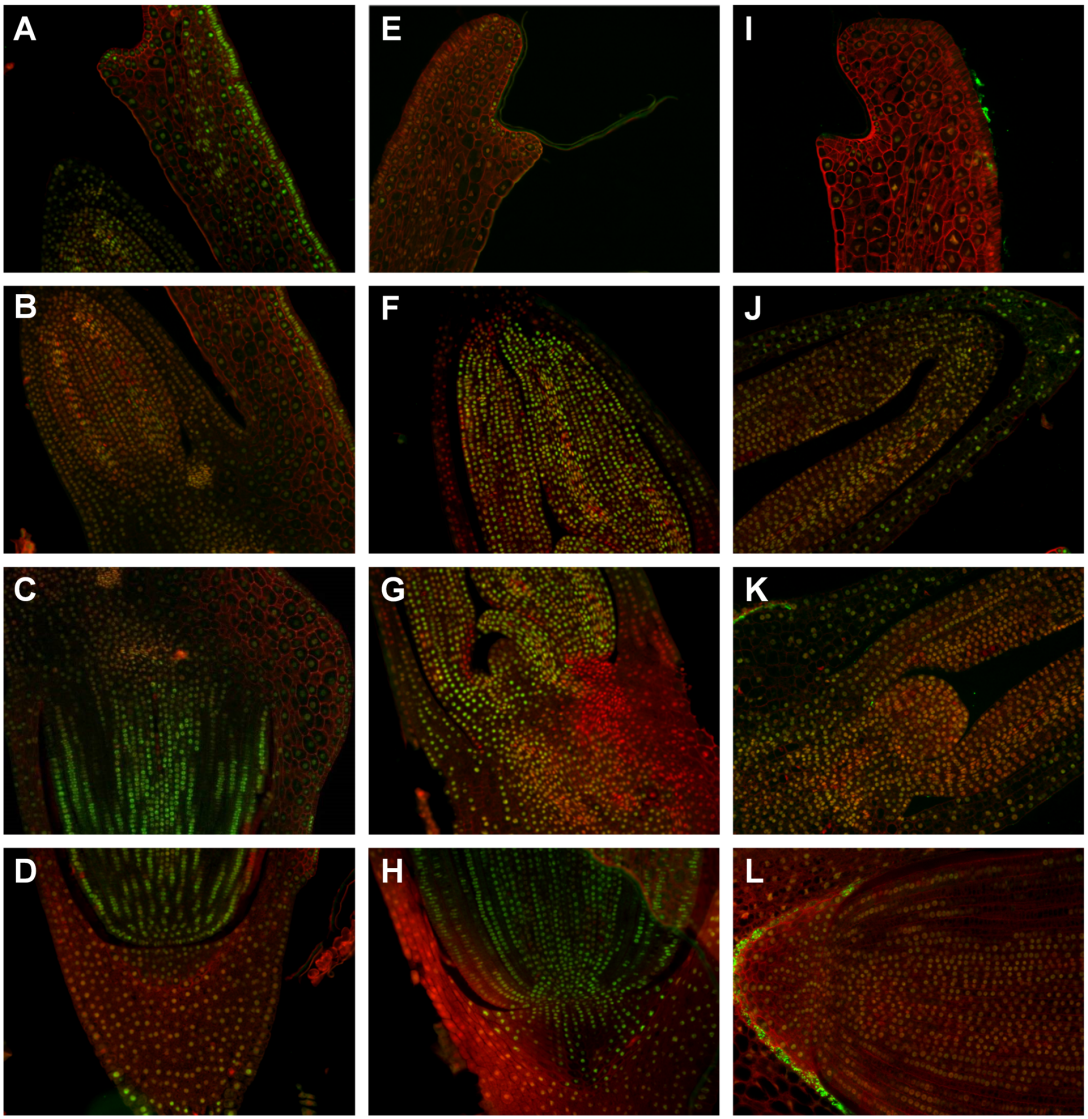
The immunodetection of H3K4me2 in ‘matured’ (A–C), ‘dry’ (D–G) and ‘germinating’ (H–K) Brachypodium embryos. Cross sections through the scutellum (**A, E, I**), the coleoptile and leaf primordia (**B, F, J**), the SAM with leaf primordia (**G, K**), the proximal part of RAM (**C**), the RAM, root cap and coleorhiza (**D, H, L**). Bar: 50 µm. Enlargements of selected cross sections are provided (Figure S1).

The seeds from which each type of Brachypodium embryo was dissected were similar in size (Figure 2). By using the PAS staining technique, a visualisation of their internal structures was possible. Longitudinal sections were excised from the middle part of the embryo and included the scutellum, coleoptile, SAM with leaf primordia, RAM with the root cap and coleorhiza (Figure 1). ‘Matured’ and ‘dry’ embryos revealed the same stage of development of both embryo types and the general shape and all of the structures of the embryos were visible (Figure 3A–3B). Embryos from germinating seeds were slightly larger than the ones derived from matured and dry seeds. During the 12 h of germination, the coleorhiza and embryo root began a rapid elongation and became vacuolated (Figure 3C). For presentation purposes on Figures 4–7, four representative images from the appropriate region (scutellum, coleoptile and leaf primordia, SAM, RAM, root cap and coleorhiza) obtained from the same embryo section were chosen for each type of epigenetic modification as well as for the detection of starch.

### Starch accumulation is most prominent in ‘germinating’ embryos

PAS staining enabled the identification of starch grains in diverse tissues of embryo. A detailed analysis revealed distinct amounts of starch grains in all of the types of embryos that were studied. The ‘dry’ embryos had the smallest amount of starch grains (Figure 4E–4H) in comparison with the ‘matured’ and ‘germinating’ embryos (Figure 4A–4D and 4I–4L). The latter ones had the highest amount of starch that was visible in almost all of the types of embryo tissue (Figure 4I–4L). A diverse number of starch grains was revealed among distinct types of embryo tissues in ‘matured’ and ‘dry’ embryos. Each embryo type contained starch in the scutellum cells but a relatively low amount of starch was observed in this area in the case of ‘dry’ embryos (Figure 4A, 4E and 4I). A lack of starch was observed in the SAM and primary leaves of ‘dry’ embryos (Figure 4G) but a few starch grains were localised in the corresponding regions of the ‘matured’ embryos (Figure 4C). The RAM cells of both embryo types did not contain starch (Figure 4D and 4H), in contrast to the ‘germinating’ ones (Figure 4L). The root cap and coleorhiza cells of the ‘matured’ (Figure 4D) and ‘germinating’ (Figure 4L) embryos contained starch grains, unlike the corresponding cells in the ‘dry’ embryos (Figure 4H).

### Histone H4 acetylation and H3 methylation in ‘matured’ embryos are most prominent in RAM

Strong immunofluorescence signals corresponding to H4K5 acetylation were visible only in nuclei from epithelial cells of the scutellum (Figure 5A). This modification was not detected in most of the coleoptile cells, contrary to the SAM and leaf primordia where it was the most abundant (Figure 5B). In the RAM, the highest intensity of signals was detected (Figure 5C), whereas in the coleorhiza cells no H4K5ac was observed (Figure 5D and Table 1). The intensity of immunosignals corresponding to another modification analysed–H3K4me1 was similar within most of the tissues of the ‘matured’ embryos. Moderate levels of this modification were detected in the nuclei of scutellar cells, SAM, RAM and leaf primordia (Figure 6A–6C). However, in coleoptiles no signals were observed (Figure 6B) and low signal intensity was detected in the nuclei of coleorhiza cells (Figure 6C and Table 1). In case of the last modification–H3K4me2, the most intense immunofluorescence was detected in the nuclei of the scutellum, especially in the epithelial cells (Figure 7A). Similarly high intensity of signals was observed in the RAM tissues (Figure 7C–7D), whereas the SAM and leaf primordia were characterised by moderate signal intensity (Figure 7B–C). The nuclei of coleorhiza and coleoptile cells revealed comparable, moderate level of this modification (Figure 7B–7D and Table 1).

**Table 1:** Relative intensity of the immunosignals in Brachypodium embryos.

Embryo type	‘Matured’			‘Dry’			‘Germinating’		
Modification type	H4K5ac	H3K4me1	H3K4me2	H4K5ac	H3K4me1	H3K4me2	H4K5ac	H3K4me1	H3K4me2
Scutellum	+	+	++	-	+	+	++	++	+
Coleoptile	−	+	+	+	++	+	−	+	++
Shoot meristem (SAM) and leaf primordia	++	+	+	++	++	++	+	++	+
Primary root (RAM)	++	+	++	+	+	++	+	++	+
Coleorhiza	−	+	+	++	−	+	−	+	+

+ and − represent, respectively, the presence or absence of immunosignals: +++, strong signal; ++, moderate signal; +, week signal.

### Histone H4 acetylation and H3 methylation in ‘dry’ embryos are most prominent in SAM and leaf primordia

H4K5ac was detected only in the cytoplasm of some scutellar cells but not in the cytoplasm of epithelial and provascular cells (Figure 5E). In most of coleoptile cells moderate signals of H4K5ac were visible (Figure 5F). In the SAM, leaf primordia (Figure 5F), distal part of RAM and coleorhiza (Figure 5G–5H) immunofluorescence was the most intense (Table 1). The intensity of immunosignals corresponding to H3K4me1 was the highest in nuclei from the coleoptile and leaf primordia (Figure 6E). Moderate intensity of immunosignals was observed in the nuclei of scutellum (Figure 6D–6E) and RAM (Figure 6F–G), while no immunofluorescence was detected in coleorhiza (Figure 6G and Table 1). The most intense signals (Table 1) of H3K4me2 were detected in leaf primordia (Figure 7F), SAM (Figure 7G) and RAM (Figure 7H), while only moderate intensity of immunosignals was observed in the scutellar cells (Figure 7E) and majority of coleoptile (Figure 7F) and coleorhiza (Figure 7H) cells.

### Histone H4 acetylation and H3 methylation in ‘germinating’ embryos are most prominent in scutellum

Each type of the scutellar cells exhibited strong immunofluorescence signals corresponding to H4K5ac (Figure 5I). Immunofluorescence was not detected in most of the coleoptile, leaf primordia (Figure 5J) and coleorhiza (Figure 5L) cells, whereas moderate signals were observed in the SAM and RAM (Figure 5J–5K and Table 1). H3K4me1 immunosignals were the most intensive in the nuclei of scutellar cells (Figure 6H), while in the SAM (Figure 6J), RAM and coleorhiza (Figure 6K) moderate immunofluorescence was detected. In the nuclei of coleoptiles (Figure 6I) only very weak or absence of signals for H3K4me1 was observed (Table 1). Immunolocalisation of H3K4me2 revealed that the most intensive signals were in the nuclei of the coleoptile (Figure 7J) and comparably high signals were found in the cells of leaf primordia and SAM (Figure 7J–K). A moderate intensity of immunosignals was detected in the scutellum (Figure 7I) and RAM (Figure 7L), whereas the lowest level was observed in the coleorhiza (Figure 7L and Table 1).

## Discussion

The seed and embryo are important stages of plant development that have an influence on the life cycle of a new plant. With the emergence of Brachypodium as a reference organism for temperate cereals, a comprehensive overview of its seed development was done by Guillon et al. [26]. As seed development after fertilisation is divided into three main phases, namely seed maturation, desiccation and storage, and seed germination, these stages were examined in our study. We used the time span of Brachypodium grain development that corresponded to the time period that was described by Guillon et al. [26] for the plant growth conditions.

Embryo development is characterised by rapid expansion growth, synthesis and accumulation of storage reserves. Arabidopsis embryos have an initial phase of starch accumulation, which takes place before the accumulation of storage oil. According to Andriotis et al. [35], starch levels decline during embryo development and are almost undetectable at maturity. These authors suggested than in oilseed species starch turnover seems to be functionally linked with cell division and differentiation rather than with developmental or storage functions. This hypothesis may also be true for Brachypodium embryos, as the lowest amount of starch granules was detected in ‘dry’ embryos, which represent a quiescent state of embryos during development. By contrast, the highest amount of starch was found in ‘germinating’ embryos, when germination processes take place and activation of cell growth and divisions occurs. Differences in starch accumulation were evident not only among the three types of embryos that were analysed but also among different tissues/organs of the same embryo type. Such an observation can indicate differences in the physiological state of the embryos as well as the role of particular organs and tissues in their development. A good example would be the scutellum, which plays a pivotal role in the absorption of nutrients from the endosperm. Although the accumulation of carbohydrates during Brachypodium seed development was described by Guillon et al. [25], [26], these authors were mainly focused on the seed as a whole, while we describe the starch accumulation in specific embryo tissues.

Plants have the remarkable ability to react to seasonal changes by synchronising their life cycle transitions with environmental conditions. The switch from one developmental phase to the following requires significant changes in both the spatial and temporal patterns of gene expression. The transcriptional reprogramming of these genes involves the active modification of their chromatin structure. Many studies have indicated that chromatin organisation is a dynamic process and that it undergoes considerable reorganisation during plant development; a good example are the alterations in the chromatin organisation and expression dynamics of 5S rDNA loci that were studied in Arabidopsis shortly after germination [36]. The large-scale reorganisation of chromatin has been attributed to developmental and environmental stimuli [37], [38] and also as the result of cellular dedifferentiation [39] or developmental transitions such as seed maturation and germination [15], seedling growth [40], [41] and floral transition [42]. Although epigenetic regulation of gene expression during most of seed developmental stages was examined [43], [44], the analyses of epigenetic modifications in maturing or especially in dry seeds, have not yet been demonstrated. This is probably due to the highly compacted state of chromatin in dry seeds, which is likely to hamper effective access of antibodies [15].

Most data concerning seed and embryo development comes from studies on Arabidopsis, which is a representative of dicotyledonous plants and was the first angiosperm model organism. In our research we studied the embryo of a monocotyledonous reference plant, Brachypodium, in an attempt to elucidate crucial embryo transition processes by tracking chromatin dynamics with particular attention being paid to the three chromatin marks–H4K5ac, H3K4me1 and H3K4me2. Most importantly, unlike other studies that were performed on Arabidopsis [15], [21] or *Z. mays* [45], we used embryo tissue sections exclusively but not isolated nuclei. Such an approach, though methodologically demanding, enables the analysis of the distinct marks of epigenetic modifications in the nuclei of particular embryo tissues and organs with their preserved topographical context. Recently, this strategy was tested and successfully applied to investigate the spatial and temporal distribution of epigenetic modifications in the root apical meristem of *Hordeum vulgare* seedlings [33], [46].

During seed maturation, seeds become dehydrated and tolerant of desiccation. At this phase storage compounds are accumulated and dormancy is induced [47]. Evidence for the involvement of histone modifications in seed maturation are scarce. One example is histone H2B monoubiquitination, which in plants is not well understood. Several dormancy-related genes show reduced levels of transcripts in *hub1* mutant seeds [48]. Recently, a role for another histone modification in seed dormancy was demonstrated. Mutations in the *KYP/SUVH4* gene, encoding the histone methyltransferase responsible for H3K9me2, caused increased seed dormancy [49]. In addition, microRNAs were shown to be important regulators of the timing of embryo maturation [50]. However, to the best of our knowledge there is no similar study demonstrating global, topographical distribution of histone epigenetic modifications during embryo maturation. Our study revealed the presence of high levels of histone H4 acetylation in the main parts (root, hypocotyl, leaf primordia) of matured embryos. It was shown that one of the histone H3 acetylases–AtGCN5 is required for the formation of embryonic root [51] and to maintain root meristem activity [52]. Histone H4 acetylation is known to be associated with euchromatin and gene transcription, so high levels of this modification can reflect the activity of genes responsible for embryo maturation, as well as genes involved in dormancy induction. The other two modifications we analysed, i.e. H3K4me1 and H3K4me2, are also typical euchromatin markers. Contrary to H4K5ac, H3K4me2 was detected at high levels in scutellar cells, particularly in the epidermis.

Dry seeds represent an intermediate state between seed maturation and germination and, in comparison with the rest of the plant life cycle, exhibit some exceptional characteristics, such as extremely low moisture contents which is often well below 10%. Several recent studies have demonstrated that vast changes at the transcript and protein levels occur in dry seeds during storage and that they might be targeted to release dormancy as seeds after-ripen [53]. This might result from hydrated pockets within cells [54], which in turn may enable various processes associated with germination [4]. Our results showed high levels of H3K4me2 in the main tissues of the ‘dry’ embryo, which may indicate that this histone modification may be linked with transcriptional activity of embryo cells during seed storage. In turn, changes in chromatin modifications require multiple enzymatic processes and it follows that active epigenetic signalling is unlikely to occur in the dry seed. However, stored seeds do show gradual changes in traits, whose effects only become evident when they are imbibed under favourable environmental conditions, thus enabling germination [53]. Additionally, dry seeds contain a large amount of stored transcripts that have been generated during seed maturation [55]. Many of these transcripts will be translated upon imbibition and have a pivotal role in germination [56]. During the ‘dry’ seed state, replication is arrested and the cells accumulate at G1 until rehydration [57]. It cannot be ruled out that histones may accumulate as a free pool in the cytoplasm when DNA replication is inhibited. Almost instantly after seed rehydration, the nuclei enter S phase, reinitiate transcription processes and repair potential DNA damage linked with the former ‘dry’ state [58]. As some desiccation-stable proteins, such as histones, are always present in their active form in the cytoplasm, this may in some way explain our observations of H4K5ac cytoplasmic signals during seed desiccation. In the scutellum of ‘dry’ embryos, contrary to ‘matured’ ones, H4K5ac signals were identified only in the cytoplasm with the exception of epithelial and provascular cells. Cytoplasmic localisation of H4K5ac immunofluorescence was also detected in some meristematic cells of *H. vulgare*, and although there are some explanations of this phenomenon from studies of human and yeast, it is not clear if they are also valid for plants [33]. Chromatin of ‘dry’ embryos, which are in a quiescent state, exhibits intensive immunofluorescence signals corresponding with transcriptionally active euchromatin. Such an observation is intriguing, as finding less intense signals or their absence would be expected in these embryos. However, the methodology-related explanation, linked with the fact that seed fixation in an aqueous solution of paraformaldehyde has some influence on the activation of processes involved in seed imbibition and therefore elevated levels of euchromatin-specific markers, cannot be ruled out.

One of the most commonly reported features of cell nuclei in dry quiescent embryos is their highly condensed chromatin [19]. In 2011, van Zanten et al. [15] reported significantly reduced nuclear size in embryonic cotyledons of Arabidopsis that were accompanied by epigenetic modifications of the chromatin. They attributed this phenomenon to the initiation of seed maturation. During germination these nuclei regain their ‘normal’ size, which is linked to chromatin decondensation. In our study such a reduction in nuclear size in dry quiescent embryos was not observed, which suggests that the Brachypodium embryo may be an exception to this rule.

Germination is defined as a protrusion of the radicle through surrounding seed tissues. It was proposed over two decades ago that change in the transcriptional programme from maturation to germination takes place during early imbibition. This highly coordinated change in gene activity is essential for germination and requires strict regulation at the epigenetic level [53], [59]. The functions of chromatin remodelling and epigenetic signalling during germination have been intensively studied [43], [60]. Recently, Müller et al. [14] elucidated an important aspect of the transition from seed dormancy to germination and seedling growth by following the chromatin dynamics of key regulatory genes with a focus on the two antagonistic marks, H3K4me3 and H3K27me3. They observed a switch from H3K4me3 and high transcription levels to silencing by the repressive H3K27me3 mark when dormancy was broken through the exposure to moist chilling, which underscores the fact that a functional PRC2 complex is necessary for this transition.

Seed germination is linked with major changes in transcript levels of dormancy- and germination-related genes. This transcriptional changes are associated with prominent changes in chromatin structure [53], which in turn are mainly directed by epigenetic modifications. Our results show that in ‘germinating’ embryos, contrary to the ‘matured’ and ‘dry’ ones, high level of H4K5ac was detected in scutellar cells, while a significantly lower level was observed in root and leaf primordia. This may indicate that the switch in transcriptional profiles of genes may be also interconnected with the switch in levels of histone modifications. The scutellum plays a key *role* in the absorption of degraded material during germination from the endosperm and transfers it to the growing embryo. Therefore elevated levels of the H4K5ac marker may reflect high metabolic activity of this tissue.

DNA methylation is another epigenetic modification that plays an important role in plant development. Experimental evidence from various species has unequivocally established its importance at least during some developmental stages. For example, cytosine methylation regulates imprinted gene expression in the endosperm thus assuring normal embryonic development in sorghum [61]. Reports on the global DNA methylation profiling of the endosperm and embryonic genomes of Arabidopsis show a widespread reduction in DNA methylation in the endosperm, particularly in regions that correspond with transposable elements and small RNAs [62], [63]. Global demethylation in the maternal endosperm genome was observed in *Oryza sativa* [64] and *Z. mays* [65]. Recently Kapazoglu et al. [66] characterised a gene encoding a DNA glycosylase that is closely related to cereal DME glycosylases in *H. vulgare*. Expression analysis during seed development and under stress conditions suggests its role in endosperm development, seed maturation and in the response to drought. We have also attempted to study two heterochromatin-specific markers, DNA methylation (5 mC) and H3K9me2 (data not shown, as these modifications do not display typical tissue-specificity). Seed desiccation is a physiological process of drying and the transition from the period of reserve accumulation to seed desiccation is associated with massive changes in gene expression. This indicates that seed desiccation is also a very active stage with respect to transcription. It is speculated that many of the biological processes occurring during seed desiccation may actually support the following germination [2].

## Conclusions

The data presented in this paper clearly demonstrate that the patterns of epigenetic modifications vary not only between particular tissues of the same embryo type but also between different types of embryos analysed. The scutellum, coleorhiza and coleoptiles are the most variable organs in terms of histone H4 acetylation and histone H3 methylation in all three types of embryos analysed. Although the involvement of epigenetic modifications of chromatin in seed development is not yet well understood, it is apparent that plants modulate their physiology and development using epigenetic mechanisms. Our results suggest that these modifications may play an important role in the organs transmitting stimuli to the embryo during seed maturation, desiccation, and germination. As this is the first study of modifications to epigenetic patterns in plant embryos in a global, topographical context and at different stages of seed development, we believe that it lays the foundations for further investigations of various epigenetic aspects of seed maturation, dormancy and germination processes.

## Supporting Information

Figure S1 **Enlargements of selected cross sections presented in** **Figure 5** **(H4K5ac) and 7 (H3K4me2).** The immunodetection of H4K5ac in ‘matured’ **(A–C)**, ‘dry’ **(D, E)** and ‘germinating’ **(F, G)** Brachypodium embryos. Cross sections through the scutellum **(A, D, F)**, the RAM **(B)** and the coleorhiza **(C, E, G).** The immunodetection of H3K4me2 in ‘matured’ **(H–J)**, ‘dry’ **(K–M)** and ‘germinating’ **(N, O)** Brachypodium embryos. Cross sections through the scutellum **(H, K)**, the SAM **(L)**, the coleoptile **(N)**, the RAM **(I)**, and the coleorhiza **(J, M, O).**
(PPTX)
