# Supplementary material for: Spatial Distribution of Epigenetic Modifications in Brachypodium distachyon Embryos during Seed Maturation and Germination
Source: PLoS One. 2014 Jul 9;9(7):e101246. doi: 10.1371/journal.pone.0101246 (PMC4090163; doi:10.1371/journal.pone.0101246)
Supplement: Figure S1 — Enlargements of selected cross sections presented in Figure 5 (H4K5ac) and 7 (H3K4me2). The immunodetection of H4K5ac in ‘matured’ (A–C), ‘dry’ (D, E) and ‘germinating’ (F, G) Brachypodium embryos. Cross sections through the scutellum (A, D, F), the RAM (B) and the coleorhiza (C, E, G). The immunodetection of H3K4me2 in ‘matured’ (H–J), ‘dry’ (K–M) and ‘germinating’ (N, O) Brachypodium embryos. Cross sections through the scutellum (H, K), the SAM (L), the coleoptile (N), the RAM (I), and the coleorhiza (J, M, O). (PPTX) [file pone.0101246.s001.pptx]

## Slide 1
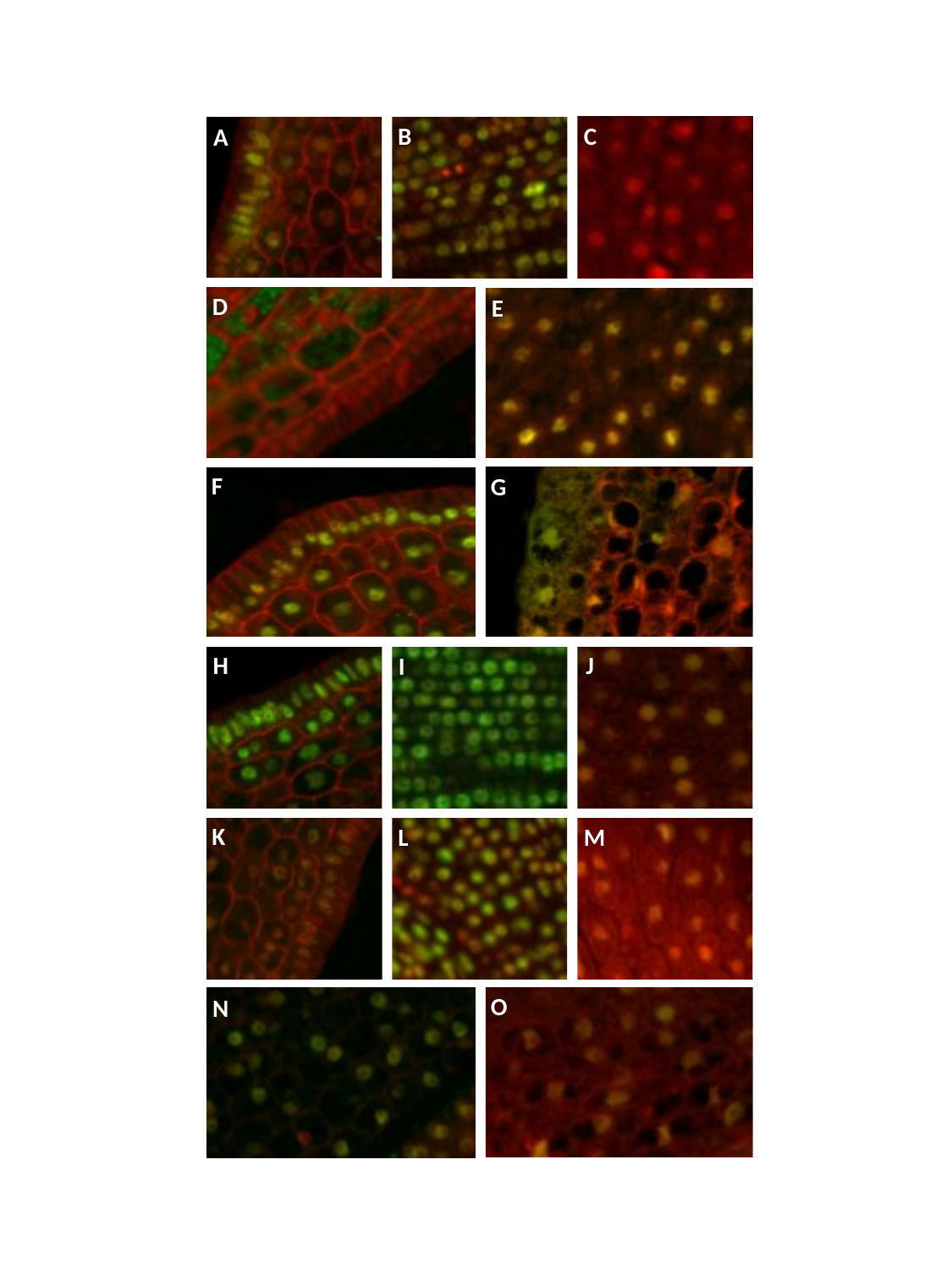

C
B
A
D
E
F
G
J
H
I
K
L
M
O
N

## Slide 2
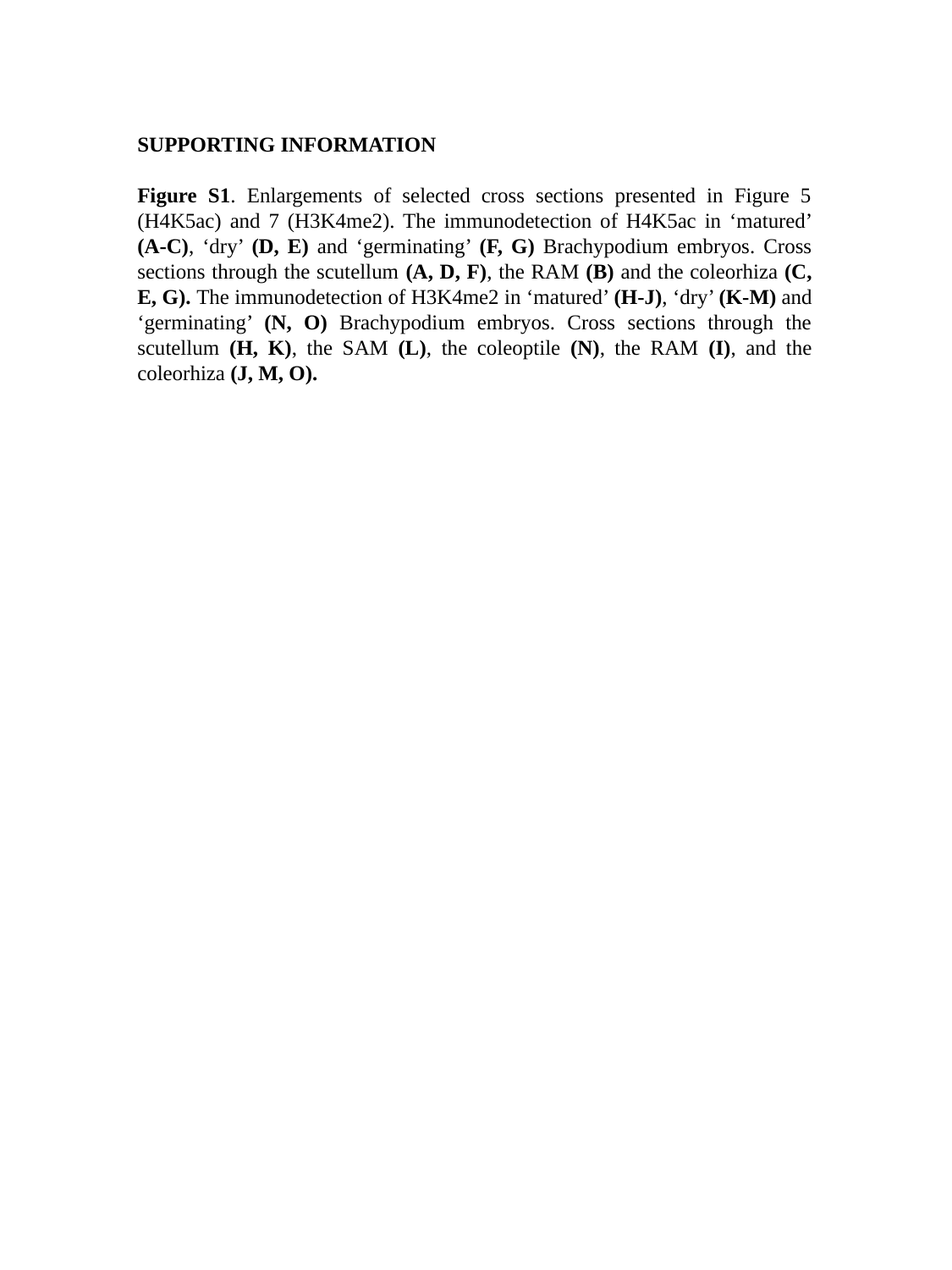

SUPPORTING INFORMATION
Figure S1. Enlargements of selected cross sections presented in Figure 5 (H4K5ac) and 7 (H3K4me2). The immunodetection of H4K5ac in ‘matured’ (A-C), ‘dry’ (D, E) and ‘germinating’ (F, G) Brachypodium embryos. Cross sections through the scutellum (A, D, F), the RAM (B) and the coleorhiza (C, E, G). The immunodetection of H3K4me2 in ‘matured’ (H-J), ‘dry’ (K-M) and ‘germinating’ (N, O) Brachypodium embryos. Cross sections through the scutellum (H, K), the SAM (L), the coleoptile (N), the RAM (I), and the coleorhiza (J, M, O).
